# Hepatic Artery Infusion of Floxuridine in Combination With Systemic Chemotherapy for Pancreatic Cancer Liver Metastasis: A Propensity Score-Matched Analysis in Two Centers

**DOI:** 10.3389/fonc.2021.652426

**Published:** 2021-04-28

**Authors:** Changli Peng, Bin Xu, Juxiong Xiao, Chunhui Zhou, Xiaodong Li, Hongbing Shi, Weiguang Qiang, Tianming Wang, Jiemin Zhao, Fei Liu, Gang Li, Haiping Li, Changyong Chen, Liangrong Shi

**Affiliations:** ^1^ Interventional Radiology Center, Department of Radiology, Xiangya Hospital Central South University, Changsha, China; ^2^ Research Center for Geriatric Disorder, Xiangya Hospital Central South University, Changsha, China; ^3^ Department of Tumor Biological Treatment, The Third Affiliated Hospital, Soochow University, Changzhou, China; ^4^ Department of Oncology, The Third Affiliated Hospital of Soochow University, Changzhou, China

**Keywords:** pancreatic cancer, liver metastasis, hepatic artery infusion, floxuridine, propensity score

## Abstract

**Aim:**

To evaluate the efficacy of hepatic artery infusion (HAI) of floxuridine (FUDR) in combination with systemic chemotherapy in patients with pancreatic cancer liver metastases (PCLM).

**Patients and Methods:**

We retrospectively collected clinical data of 347 patients with PCLM who underwent first-line chemotherapy at two Chinese centers between 2012 and 2019. Propensity score matching between patients with and without HAI was performed to compensate for differences in baseline characteristics. Objective response rate (ORR) and overall survival (OS) between groups were compared. HAI pump functionality was recorded.

**Results:**

Data of 258 patients (62 patients with HAI and 196 patients without HAI) were used for matching. After 1:1 ratio matching, 62 patients per group were included. The intrahepatic ORR was 66.1% in the HAI group and 22.6% in the non-HAI group (*P* < 0.001), and the extrahepatic ORR was 25.0 *versus* 28.9% (*P* = 0.679). The median OS was significantly longer in HAI group (14.0 *versus* 10.8 months, *P* = 0.001). Multivariance COX regression showed HAI led to a decrease in hazard ratio for death by 61.8% (HR = 0.382; 95% CI: 0.252–0.578; *P*< 0.001). Subgroup analysis revealed that patients without EHM, with higher intrahepatic tumor burden and with synchronous liver metastasis benefited more from HAI. Dysfunction of HAI pump occurred in 5.7% of patients during the period of follow-up.

**Conclusions:**

In patients with PCLM, first-line treatment with HAI FUDR plus SCT resulted in higher intrahepatic response and better OS.

## Introduction

Pancreatic cancer is the seventh leading cause of global cancer deaths in industrialized countries (1). The majority of patients present with locally advanced or metastatic disease at initial diagnosis (2, 3), which leads to poor prognosis and a 5-year survival at 7% (4). Although the clinical outcome is still limited, chemotherapy remains the primary treatment modality for patients with metastatic pancreatic cancer (5). Given liver metastasis is most frequent situation and primarily responsible for the high mortality of pancreatic cancer (6), robust management of intrahepatic lesions may provide survival benefit.

Hepatic artery infusion (HAI) chemotherapy provides high drug exposure of the tumor at first passage, which offers theoretical advantages over systemic administration of drugs (7, 8). Floxuridine (FUDR), a deoxyribonucleoside derivative of 5-Fu, is an ideal agent for HAI due to its short half-life and extensive first pass extraction (9). Commonly, FUDR was administered continuously at low dose *via* HAI pump in combination with standard systemic chemotherapy (SCT). We and others have demonstrated that HAI plus SCT is effective in improving hepatic response and prolonging survival in colorectal cancer liver metastases (CRCLM) (10–13). Recently, HAI FUDR was also demonstrated to be effective in intrahepatic cholangiocarcinoma (IHC) (14).

Herein, we evaluate the efficacy of HAI FUDR *via* a radiologically implanted pump in patients who had pancreatic cancer liver metastases (PCLM) in two centers. The primary aim was to compare overall survival (OS) from HAI plus SCT *versus* SCT alone. The second aims were to evaluate the short-term effect and functionality of HAI pump system.

## Patients and Methods

### Patients

From 2012 to 2019, consecutive histologically confirmed PCLM patients who received first-line chemotherapy were included from prospectively maintained databases in two Chinese centers (Xiangya Hospital, Central South University; the Third Affiliated Hospital of Soochow University). Patients with metachronous metastasis were included if the interval between the occurrence of liver metastasis and the end of adjuvant chemotherapy is more than 6 months. Patients who received concurrent radiotherapy were excluded. The cohort was divided into two groups according to whether the patients received HAI FUDR therapy. This study was approved by the Medical Ethics Committee of the Xiangya Hospital and the Ethics Committee of the Third Affiliated Hospital of Soochow University.

The following clinical and pathologic data were collected: age, gender, performance status, primary tumor location, time to liver metastasis, the number of liver lesion, presence of extrahepatic metastasis, baseline level of serum CA19-9, diabetes, chemotherapy regimen, objective response (assessed with CT-enhanced scan according to RECIST criteria version 1.1), and adverse events related to HAI pump system.

### SCT and HAI

SCT regimens were determined by the oncologist on the basis of guidelines, chemotherapy history, and physical conditions of patients. Gemcitabine monotherapy, gemcitabine based dual drug regimen, and triple drug regimen FOLFIRINOX were acceptable.

HAI pump system (Celsite, B. Braun, Chasseneuil, France) was implanted under digital subtraction angiography (DSA) guidance using “side-hole” and “tip-fixation” technology as previously described (12, 15). The extrahepatic branches such as the right gastric artery and the dorsal pancreatic artery were embolized to prevent extrahepatic perfusion. When multiple arteries are involved in the liver blood supply, the catheter is placed in the dominant artery, and the non-dominant branches are embolized to ensure whole hepatic infusion.

FUDR was administrated immediately after HAI pump implantation, at 0.15 mg/kg/day with dexa-methasone (DXM) at 1 mg/m^2^/day and low molecular heparin 3,200 U in saline, which lasted for 14 days respectively as described previously (16). This type of HAI regimen was administered by a Baxter infusor. SCT was started concurrently with HAI.

Dose reduction for systemic chemotherapy was made in the event of toxicity, which was assessed according to the National Cancer Institute-Common Terminology Criteria for Adverse Events (NCI-CTCAE) version 3.0. If an ulcer or gastro-duodenitis was documented, HAI therapy was held for 1 month to allow healing and the dosage of FUDR and DXM was reduced by 50% in subsequent therapies. The HAI therapy was terminated if intrahepatic progression was recorded or technical catheter-related problems and excessive toxicity related to HAI occurred.

### Statistical Analysis

The primary aim, overall survival (OS), was defined as the time from diagnosis of liver metastasis to death or last follow-up. The secondary aims were objective response rate (ORR) and functionality of HAI pump.

A propensity score was computed using a multivariable logistic regression model, with the treatment groups as the dependent variables and potential confounding factors as covariates. The following five variables were included in the propensity score matching: age, female, number of liver metastasis, synchronous liver metastasis, and with extrahepatic metastasis. All patients in the HAI group were matched 1:1 to patients in the non-HAI group, as reported previously.

Distribution difference of categorical variables was compared using Fisher’s exact or χ^2^ test. A Mann–Whitney test was used for intergroup comparisons of continuous variables. Survival curves were compared using the log-rank test. Cox proportional hazards regression model was used to adjust for age, gender, SCT regimen, number of liver lesion, time to hepatic metastasis, CA19-9 level, and presence of extrahepatic liver metastasis (EHM).

All statistical analyses were performed using the SPSS 22.0 software (SPSS Inc., Chicago, IL, USA). *P <*0.05 was considered statistically significant.

## Results

### Patients

From 2012 to 2019, 347 patients with PDLM received first-line chemotherapy in Xiangya Hospital of Central South University and the Third Affiliated Hospital of Soochow University. Among them, 28 patients who had underwent chemoradiotherapy were excluded from this study. In addition, 61 patients (including 26 with obstructive jaundice and 35 with peritoneal carcinomatosis at baseline) receiving SCT alone were excluded for there were no matched patients in the HAI group. Finally, 258 patients (62 patients with HAI and 196 without HAI) were included. **Table 1** lists the comparisons of baseline characteristics between the HAI and non-HAI groups. In the whole population, there were significant differences in number of liver lesion, EHM, and CA19-9 level between the two groups. In the HAI group, patients who had liver lesions >10 and/or CA19-9 >800 ng/ml were significantly more than those in the unmatched non-HAI group. In contrast, more patients had EHM in the unmatched non-HAI group. After 1:1 ratio matching, 62 patients per group were included. The two groups were well matched in terms of age, gender, PS score, time to liver metastasis, and EHM. However, observed but not statistically significant difference existed in the number of liver lesion (*P* = 0.104) and CA19-9 level (*P* = 0.151).

**Table 1:** Baseline clinical data.

Characteristic	All patients			Matched patients		
	non-HAI (n=196) (%)	HAI (n=62) (%)	*P* value	non-HAI (n=62) (%)	HAI (n=62) (%)	*P* value
Age [median(range)]	63.4 (38–75)	62.5 (42–75)	0.443	63.1 (38–75)	62.5 (42–75)	0.681
Age≥65	83 (42.3)	26 (41.9)	0.954	26 (41.9)	26 (41.9)	1.000
Female	88 (44.9)	24 (38.7)	0.392	24 (38.7)	24 (38.7)	1.000
ECOG score			0.154			0.983
0	76 (38.8)	28 (45.2)		27 (43.5)	28 (45.2)	
1	98 (50.0)	32 (51.6)		33 (53.2)	32 (51.6)	
2	22 (11.2)	2 (3.2)		2 (3.2)	2 (3.2)	
Tumor location			0.311			0.607
Head	78 (39.8)	18 (29.0)		23 (37.1)	18 (29.0)	
Body	64 (32.6)	24 (38.7)		20 (32.3)	24 (38.7)	
Tail	54 (27.6)	20 (32.3)		19 (30.6)	20 (32.3)	
SCLM	117 (59.7)	39 (62.9)	0.593	39 (62.9)	39 (62.9)	1.000
No. of LM			0.008			0.104
<10	112 (57.1)	23 (37.1)		33 (53.2)	23 (37.1)	
≥10	84 (42.9)	39 (62.9)		29 (46.8)	39 (62.9)	
EHM	82 9 (41.8)	16 (25.8)	0.006	16 (25.8)	16 (25.8)	1.000
CA19-9 (U/ml)			0.026			0.151
>800	120 (61.2)	28 (45.2)		36 (58.1)	28 (45.2)	
≥800	76 (38.8)	34 (54.8)		26 (41.9)	34 (54.8)	
Diabetes	87 (44.4)	27 (43.5)	0.908	30 (48.4)	28 (45.2)	0.719
ACT	65 (35.7)	16 (25.8)	0.277	19 (30.6)	16 (25.8)	0.549
SCT			0.113			0.407
GEM	10 (5.1)	4 (6.5)		3 (4.8)	4 (6.5)	
GC	68 (34.7)	32 (51.6)		23 (37.1)	32 (51.6)	
GEMOX	41 (20.9)	14 (22.6)		16 (25.8)	14 (22.6)	
GEMNP	45 (23.0)	8 (12.9)		15 (24.2)	8 (12.9)	
FOLFIRINOX	25 (12.8)	4 (6.5)		5 (8.1)	4 (6.5)	

ECOG, Eastern Cooperative Oncology Group; SCLM, synchronous liver metastasis; LM, liver metastasis; EHM, extrahepatic metastasis; ACT, adjuvant chemotherapy; SCT, systemic chemotherapy; GME, gemcitabine monotherapy; GBDC, gemcitabine-based dual-drug chemotherapy.

### ORR in the Matched Population

**Figure 1:**
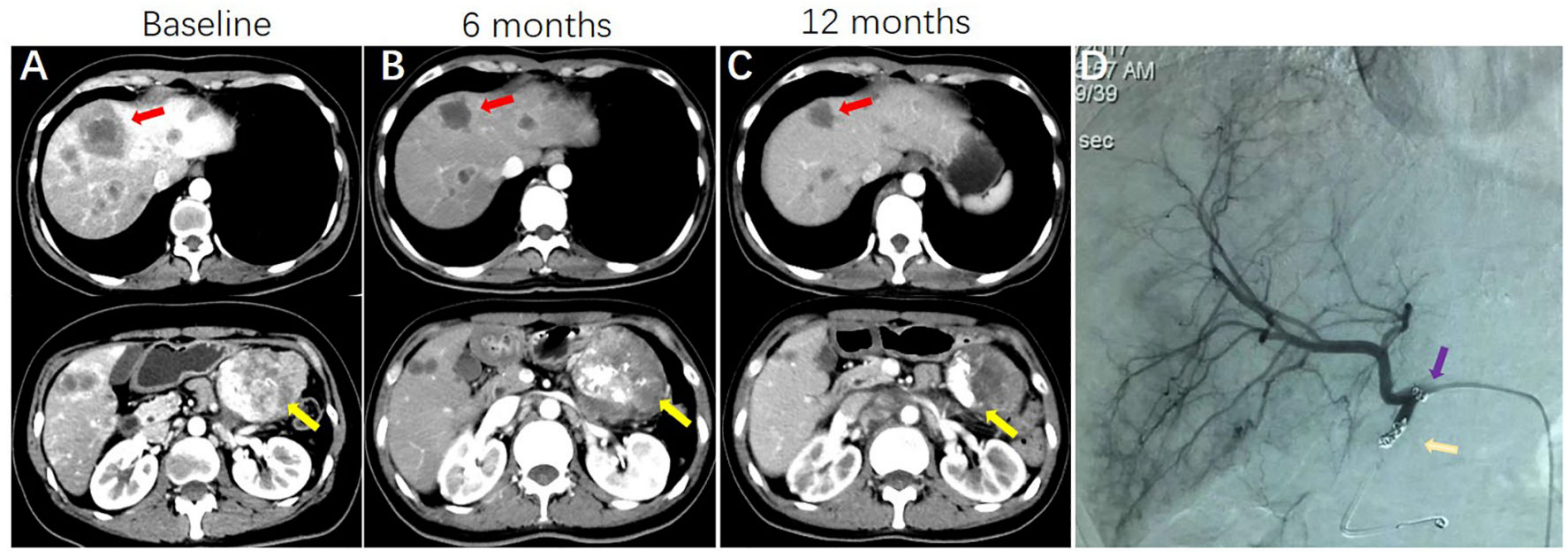
A patient with pancreatic cancer and synchronous liver metastases, who was treated with systemic GC HAI FUDR. **(A)** CT images showing the presence of pancreatic cancer (yellow arrows indicate) and synchronous liver metastases (red arrows indicate) at baseline. **(B)** CT images show primary cancer and liver metastases are shrinking at 6 months. **(C)** images show sustained response of liver metastases and primary cancer. **(D)** Hepatic arteriography shows HAI pump functionality at 12 months (purple arrow indicates side hole of the catheter; gold arrow indicates the tip of the catheter, which was located in the occluded gastroduodenal artery).

**Table 2** lists the objective response in intrahepatic and extrahepatic lesions separately. For intrahepatic lesions, five CR (8.1%) and 36 PR (58.1%) were observed in patients treated with HAI. The intrahepatic ORR was 66.1% in the HAI group, which was significantly higher than 22.6% (0 CR and 14 PR) in the non-HAI group (*P* <0.001). In the HAI group, 44 patients had evaluable extrahepatic lesions (including intact primary tumor and extrahepatic metastasis) and 11 patients (25.0%) achieved PR. In the non-HAI group the extrahepatic ORR was 28.9% (13/45). There was no significant difference in extrahepatic ORR (*P* = 0.679). **Figure 1** demonstrates CT and DSA images of a patient who achieved PR with SCT and HAI.

**Table 2:** Objective response rate n (%).

	Extrahepatic lesion*			Intrahepatic lesions		
	non-HAI (n=45)	HAI (n=44)	*P* value	non-HAI (N=62)	HAI (N=62)	*P* value
ORR	13 (28.8)	11 (25.0)	0.679	16 (25.8)	41 (66.1)	<0.001
CR	0	0	–	0	5 (8.1))	0.057
PR	13 (28.8)	11 (25.0)	0.679	16 (25.8)	36 (58.1)	<0.001
SD	16 (35.6)	19 (43.2)	0.461	24 (38.7)	19 (29.0)	0.451
PD	16 (35.6)	14 (31.8)	0.709	22 (35.5)	2 (3.2)	<0.001

*evaluation for unresectable primary cancer and extrahepatic metastases.

ORR, overall response rate; CR, complete response; PR, partial response; SD, stable disease; PD, progressive disease.

Thirty-six patients (58.1%) in the HAI group and 39 patients (62.9%) in the non-HAI group underwent second-line SCT (*P* = 0.714). In the HAI group, HAI was continuously administrated for median four cycles (range 2–7) in combination with second-line SCT in 17 patients who developed extrahepatic disease progression but had disease control in the liver. In addition, eight patients received HAI (median four cycles, range 2–6) in second-line treatment in the non-HAI group.

### OS in the Matched Population

**Figure 2:**
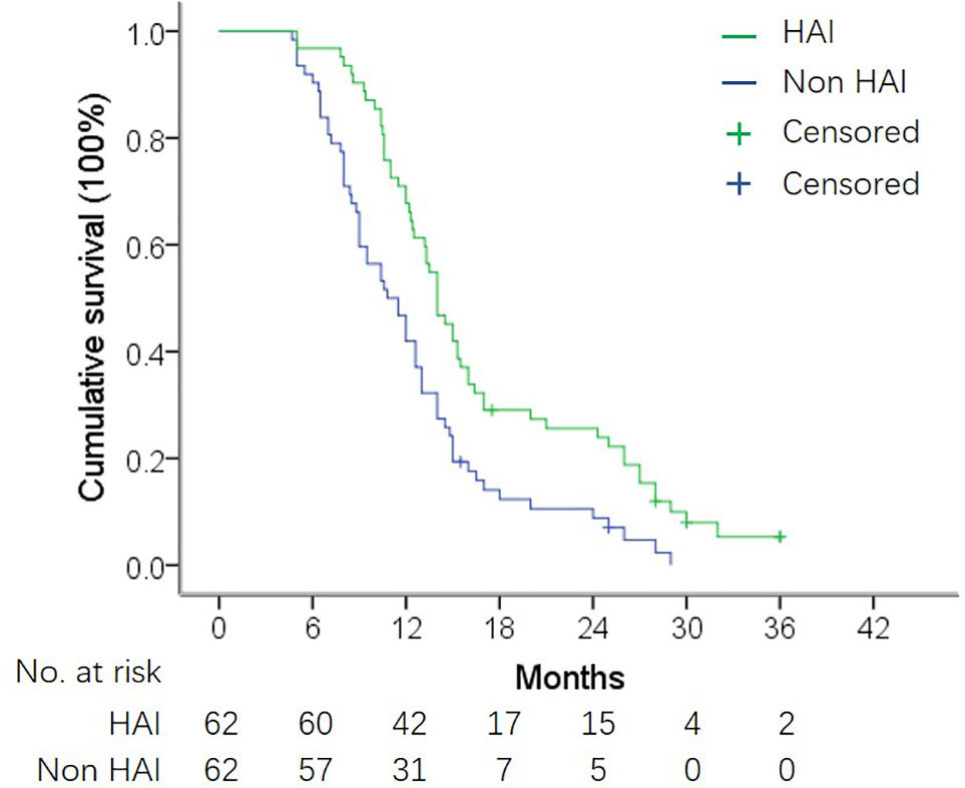
OS in matched HAI and non-HAI group. Median OS was 14.0 months *vs.* 10.8 months in patients with and without HAI (log-rank test, *P* = 0.001).

By the end of follow-up, 57 patients (91.9%) in the HAI group and 60 patients (95.2%) in the non-HAI group died. The 1- and 2-year survival rate was 71.0 and 24.2% in the HAI group and 46.8 and 9.7% in the non-HAI group, respectively. Median OS was 14.0 months (95% CI: 12.5–15.5) for patients treated with HAI and 10.8 months (95% CI: 8.7–12.9) for patients without HAI. OS for patients with HAI was better than patients without HAI (*P* = 0.001) (**Figure 2**). After adjustment for age, gender, SCT regimen, number of liver lesion, time to hepatic metastasis, CA19-9 level, and presence of EHM, HAI led to a decrease in hazard ratio for death by 61.8% (HR = 0.382; 95% CI: 0.252–0.578; *P* <0.001).

**Figure 3:**
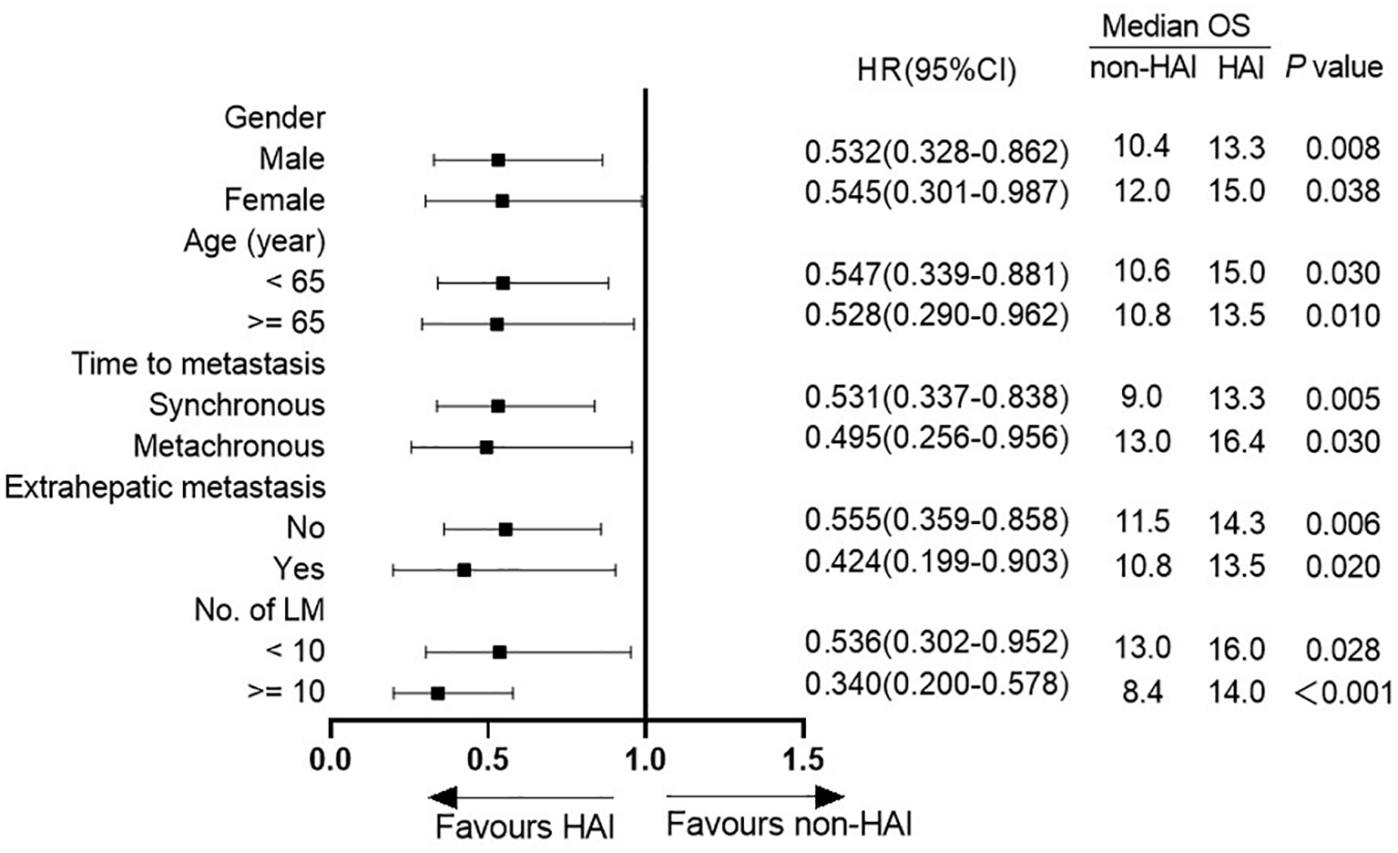
Forest plot of subgroup analysis.

**Figure 4:**
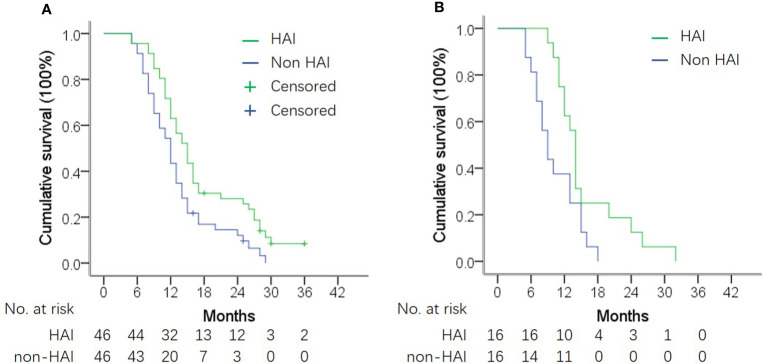
OS in subgroup analysis according to presence of extrahepatic metastasis (EHM). **(A)** OS in patients without EHM. Median OS was 14.3 *vs.* 11.5 months in patients with and without HAI (log-rank test, *P* = 0.006). **(B)** OS in patients with EHM. Median OS was 10.8 *vs.* 13.5 months in patients with and without HAI (log-rank test, *P* = 0.020).

**Figure 3** shows the difference in median OS between patients with or without HAI by subgroup analysis. HAI was associated with a better OS in all subgroups including patients with extrahepatic metastasis (**Figure 4**). Notedly, the difference was greater in subgroups without EHM, with higher tumor burden in the liver with synchronous liver metastasis. In these subgroups, HAI led to an increase of median OS more than 4 months.

### HAI Pump Implantation and Functionality

The HAI pump system was implanted successfully in all 70 patients (including eight patients who received HAI in a second-line treatment). Four patients (5.7%) discontinued HAI due to dysfunction of HAI pump. Catheter occlusion occurred in one patient after three cycles of HAI therapy. One patient had hepatic artery occlusion at 11 months. Two patients developed local abscess at the pump implant site, and the pumps were removed at 4 and 7 months. It should be noted that these two patients had insulin-resistant diabetes. No patient discontinued HAI due to liver injury caused by FUDR.

## Discussion

In the last decade, HAI was repositioned as part of a comprehensive treatment for advanced liver tumors (17), and administrated in combination with standard systemic therapy. In this study, we showed that HAI FUDR in combination with SCT was associated with an improvement in survival in patients with PCLM. The median OS was 14.0 months for patients treated with HAI as compared with 10.8 months for patients without HAI after propensity score matching (*P* = 0.008). Although the extrahepatic ORR was similar, the intrahepatic ORR was significantly higher in the HAI group (66.1 *vs* 25.8%, *P* <0.001).

In this cohort, patients with liver-predominant metastases were more likely to accept HAI therapy. Over 60% of patients receiving HAI had more than 10 liver lesions. In contrast, less patients with EHM were treated with HAI. In addition, a higher serum CA19-9 level was found in HAI-treated patients. Due to the significant imbalance of these radical factors between patients with and without HAI, propensity score matching analysis was introduced in this study. In order to reduce the number of confounding variance, patients with obstructive jaundice or peritoneal carcinomatosis, which is associated with poor prognosis in pancreatic cancer (3, 18), were excluded from the propensity score matching because they were not selected for HAI in this study. Following matching, observed differences in the number of liver lesions and CA19-9 level still existed between the two groups. In addition, the distribution of SCT regimens was not completely balanced. To eliminate the potential impacts of these radical confounders, we established multivariance Cox regression model and confirmed that HAI reduced the risk of death by 61.8% (HR = 0.382; *P* < 0.001).

Nowadays, GEM/nab-paclitaxel and FOLFIRINOX are preferred regimens for patients with locally advanced or metastatic pancreatic cancer. A randomized phase III study on GEM plus nab-paclitaxel showed that the ORR was 23% in patients with advanced pancreatic cancer (19). In 2013, another phase III study showed that 31.6% of patients achieved PR after treatment with more intensive regimen FOLFIRINOX (20). In addition, the ORR varied from 15 to 30% in patients treated with other recommended regimens, for example, GEM monotherapy, GC and GEMOX, in previous studies (21–24). As listed above, the efficacy of SCT for advanced pancreatic cancer is still unsatisfactory. In the present study, most patients (87.1% in non-HAI group and 93.5% in HAI group) received GEM-based dual-drug combinations. In the non-HAI group, the ORR was 25.8%. This result was consistent with published data on modern SCT. In the HAI group, the extrahepatic ORR was similar to that of the non-HAI group, suggesting HAI with low-dose of FUDR does not help to improve extrahepatic response.

In this study, a low dose of FUDR was administrated continuously *via* HAI pump. FUDR is a suitable agent for HAI due to its unique characteristics as compared with 5-Fu, such as shorter half-life, higher systemic clearance rate, and higher liver extraction rate (25). We and others reported HAI rapidly reduced tumor burden and promoted more than 50% of unresectable CRCLM to convert into resectable disease (12, 26, 27). Recently, HAI was reported to have efficacy in IHC, a more progressive and refractory disease as compared with CRCLM. In a single arm phase II study, 58% of patients achieved and sustained PR on treatment with HAI FUDR plus systemic GEMOX (14). Four patients (10.5%) underwent resection after treatment. In the present study, 97.8% of patients (60/62) achieved intrahepatic disease control, including 67.7% PR and 8.1% CR. Although no patients underwent resection, the sustained intrahepatic response helped to prevent liver-related complications, which may immediately threaten the patient’s life (7, 14). This may explain why patients with extensive liver metastases achieved greater survival advantage in the HAI group, and patients with extrahepatic metastasis also benefit from HAI.

In this study, HAI pump system was implanted by two different teams, with modified radiological techniques as described previously (15). Similar catheter functionality was achieved in the two centers. HAI pump dysfunction occurred in four patients (5.7%), including one catheter occlusion, one hepatic artery occlusion, and two infections, but none of them happened within the first 3 months. For HAI therapy, many research studies prefer surgery rather than radiological procedure to implant HAI pump system (11, 14), due to concerns about frequent dislodgement and extrahepatic infusion related to radiologically implanted catheter. In fact, these shortcomings of radiological procedure may be overcome by introducing modified techniques (12, 15, 28, 29). First, the rate of catheter displacement could be reduced to less than 5% by “tip-fix” technique (28, 30). In addition, under the guidance of DSA, extrahepatic branches can be accurately identified and embolized so as to reduce complications related to extrahepatic infusion (15). In addition, radiological implantation is performed under local anesthesia with minimal trauma, which allows the planned HAI and SCT to start immediately. This is a notable advantage over surgical procedure, especially for patients with progressive disease in the liver.

This study has several limitations. Foremost, it was designed as a retrospective observational study. The data were collected from the prospectively maintained databases with good integrity, which is favorable for propensity matching analysis. However, several factors that can influence survival did not reach complete matching, due to the relatively small size of the whole population. Furthermore, the distribution of SCT regimen was not well balanced (*P* = 0.407). The proportion of patients receiving GC in the HAI group was relatively higher (51.6 *vs.* 37.1%). Although the Cox model analysis confirmed that HAI significantly reduced the risk of death after adjustment for confounding factors including SCT regimen, the limitation of no definite SCT regimen should not be ignored.

## Conclusions

HAI FUDR was associated with higher intrahepatic ORR and longer OS patients with PCLM. However, this is a retrospective study with a relatively small sample size. The superiority of HAI in combination with standard SCT required further justification by multi-center randomized studies.

## Data Availability Statement

The raw data supporting the conclusions of this article will be made available by the authors, without undue reservation.

## Ethics Statement

The studies involving human participants were reviewed and approved by the Medical Ethics Committee of the Xiangya Hospital and the Ethics Committee of the Third Affiliated Hospital of Soochow University. Written informed consent for participation was not required for this study in accordance with the national legislation and the institutional requirements.

## Author Contributions

All authors listed have made a substantial, direct, and intellectual contribution to the work, and approved it for publication.

## Funding

This work was supported by grants from the National Natural Science Foundation of China (No. 81773234) and Scientific Research Project of Hunan Health and Health Commission (No. C2019189).

## Conflict of Interest

The authors declare that the research was conducted in the absence of any commercial or financial relationships that could be construed as a potential conflict of interest.
